# Serenoa Repens: Does It have Any Role in the Management of Androgenetic Alopecia?

**DOI:** 10.4103/0974-2077.53097

**Published:** 2009

**Authors:** Sundaram Murugusundram

**Affiliations:** *Consultant Dermatologist, Chennai, Tamil Nadu, India*

**Keywords:** Androgenetic alopecia, serenoa repens, 5 alpha reductase

## Abstract

Serenoa repens is one among the many naturally occurring 5 alpha reductase (5aR) inhibitors which has gained popularity as a magical remedy for androgenetic alopecia. It is widely advertised on the web and sold by direct marketing. Used as a self-medication, there is a risk of missing the early detection of prostate cancer. There is little evidence to support its efficacy, warranting larger clinical trials on androgenetic alopecia.

## INTRODUCTION

Many plant extracts claimed to have an inhibitory effect on the enzyme 5 alpha reductase (5aR) are widely advertised and sold on the internet as magic remedies for benign prostatic hypertrophy and androgenetic alopecia (AGA). Extract of serenoa repens (SR) is the most popular among the botanically derived 5aR inhibitors. Despite lack of proper clinical trials to support their efficacy, these products enjoy popularity among patients. They are also advocated by the so-called “trichologists” who promote the drug as a “safer” alternative to finasteride.

## SOURCE AND CONTENTS OF SERENOA REPENS

**Figure 1:**
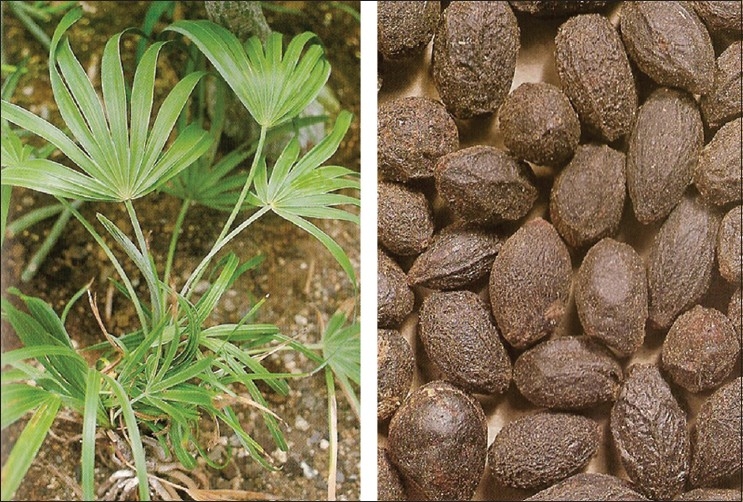
Serenoa repens - The plant and the berries

Saw palmetto is an extract from the berries of the palm tree saw palmetto (also called serenoa repens, serenoa serrulata or sabal serrulata). The plant is a native of West Indies and is grown in plenty on the Atlantic southeast coast of North America. It is a tall tree of 6 to 10 feet with a crown of thorn-shaped leaves arranged like a fan. The berries are oblong in shape and maroon colored [Figure 1]. The extract of these berries is easily available and inexpensive.

The purified extract of SR contains 85-90% fatty acids and sterols, with an abundance of carotenoids, lipases, tannin, sugars and fatty acids like caprylic acid, palmitic acid, oleic acid and beta sitosterol. The combination of saw palmetto's liposterolic extract (LSESr) and beta sitosterol has been claimed to give improvement in AGA.

## ACTIONS OF SERENOA REPENS

The mechanism of action is thought to be similar to finasteride i.e., blocking 5aR. Also, SR is thought to decrease dihydrotestosterone (DHT) uptake by hair follicle and decrease the binding of DHT to androgenetic receptors.

## USES OF SERENOA REPENS

Saw palmetto extracts have been used anecdotally for a number of indications:

1. In baldness and prostate enlargement.
2. To help build and strengthen tissue and increase metabolism.
3. As a diuretic which improves urinary flow.
4. As an expectorant to relieve chronic bronchitis, asthma and chest congestion.
5. In thyroid disorders.
6. To stimulate appetite, digestion and absorption of nutrients.
7. In polycystic ovary syndrome.

### Use in androgentic alopecia

Very few studies exist to support the claims of its efficacy. In a small study of 10 males with AGA (23-64 years) on oral SR, improvement was seen in 60%.[1] In a study of 34 men and 28 women (18-48 years) topically applied SR extract in lotion and shampoo base for three months led to 35% increase in hair density and 67% increase in sebum reduction assessed by sebometry, pH metry, hydration studies and phototrichogram (study presented at the fourth intercontinental meeting of hair research societies, June 17-19, 2004).[2] Addition of extract of 0.5% SR to ketaconazole shampoo was shown to give better results compared to ketaconazole alone (presented at the 13th Annual meeting of the European Hair Research Society, Genoa, Italy).[3]

## SIDE-EFFECTS

Side effects are said to be uncommon. The most common side effects is mild stomach discomfort which can be alleviated by taking it after food. Like other 5 alpha reductase inhibitors, SR may reduce PSA levels by 50% after 6 – 12 months of treatment. There is thus a risk of missing early detection of prostate cancer in patients self medicating with serenoa repens through the internet and direct marketing.

## FORMULATIONS

There are two types of herb palmetto supplements available in the market. One type is dried saw palmetto berries and the other type saw palmetto extract in the form of tablets. Recommended dose is 160mg twice a day. Although saw palmetto is listed in the US pharmacopoeia, it falls under the guidelines for food supplements. It is not regulated by the Federal drug authority FDA.

## CONCLUSION

Significant evidence for advocating the use of the drug in AGA is lacking. However, physicians need to be aware of the drug as it is being used by patients and opinion will be sought about its efficacy.
